# 

**Published:** 1875-04

**Authors:** 


					﻿Volume XIII. j	APRIL—1875.	Number 1.
ATLANTA
Medical and Surgical Journa ,
t
. MONTHLY: THREE DOLLARS PER ANNUM, IN ADVANCE.
EDITORS;
!	IJ()BI£irr P,ATTEY, M.l).,
i
AND
AV. F. AVESTMORELANI), AT.U.
I	PAX ET SCIENTIA, SEP VEUITAS SINE TIMOUE.
I
I
1	ATLANTA, GEORGIA:
DUNLOP, WYNNE £ CO., LOOK AND JOIi PRINTERS.
I	«
				

